# Codeposited Bimetallic Pt–Pd Catalyst Supported on MWCNTs/Carbon Cloth as an Efficient DFAFC Anode Material

**DOI:** 10.1021/acssuschemeng.5c02346

**Published:** 2025-06-18

**Authors:** Karol Juchniewicz, Izabela S. Pieta, Bogusław Mierzwa, Marcin Pisarek, Ravishankar G. Kadam, Olena Mozgova, Marcin Holdynski, Artur Malolepszy, Andrzej Borodzinski, Piotr Pieta

**Affiliations:** † 119463 Institute of Physical Chemistry, Polish Academy of Sciences, Kasprzaka 44/52, 01-224 Warsaw, Poland; ‡ Regional Centre of Advanced Technologies and Materials, Czech Advanced Technology and Research Institute (CATRIN), Palacký University Olomouc, Šlechtitelů 241/27, 783 71 Olomouc-Holice, Czech Republic; § Nanotechnology Centre, Centre for Energy and Environmental Technologies, VŠB−Technical University of Ostrava, 17. Listopadu 2172/15, 708 00 Ostrava-Poruba, Czech Republic; ∥ Warsaw University of Technology, Faculty of Chemical and Process Engineering, Warynskiego 1, 00-645 Warsaw, Poland

**Keywords:** electrodeposition, palladium−platinum catalysts, single fuel cell, DRIFT, formic acid

## Abstract

Direct formic acid fuel cell (DFAFC) is a promising energy source for portable devices due to its high theoretical open-circuit voltage (1.45 V), high power density, and the use of a nearly nontoxic fuel. To make DFAFC commercially feasible, it is necessary to develop an efficient catalyst for formic acid (FA) electrooxidation. Here, we present a nanostructured catalyst based on the Pd_0.64_Pt_0.36_ nanoparticles (Pd_0.64_Pt_0.36_ NPs) immobilized onto a carbon cloth-supported MWCNTs surface. The Pd_0.64_Pt_0.36_ NPs were electrochemically formed under potentiodynamic conditions by using linear sweep voltammetry (LSV) by electroreduction of PtCl_4_ and Pd­(OAc)_2_ precursors, previously immobilized inside the MWCNTs framework. The resulting catalyst forms ∼4 nm diameter spherical NPs, well-separated from each other and uniformly decorating the entire MWCNTs surface. XRD analysis showed the presence of Pd- and Pt-rich phases, while DRIFT measurements clearly indicate that the catalyst is resistant to CO poisoning and much more active compared to pure Pd and Pt metal catalysts. The Pd_0.64_Pt_0.36_ catalyst has a high ECSA value (56.94 m^2^/g) and at least 80% active site availability. These parameters explain its high activity and stability toward FA electrooxidation. The performance of the Pd_0.64_Pt_0.36_ catalysts as an anode was evaluated in a DFAFC cell at a temperature of 60 °C and cathodic airflow of 200 mL/min. A long-term stability study measured under a 50 mA/cm^2^ load (14 h) for 3 M HCOOH showed excellent durability of the catalyst. DFAFC with a Pd_0.64_Pt_0.36_ catalyst shows an excellent power density value of 64 mW/cm^2^ at 250 mA/cm^2^.

## Graphical abstract

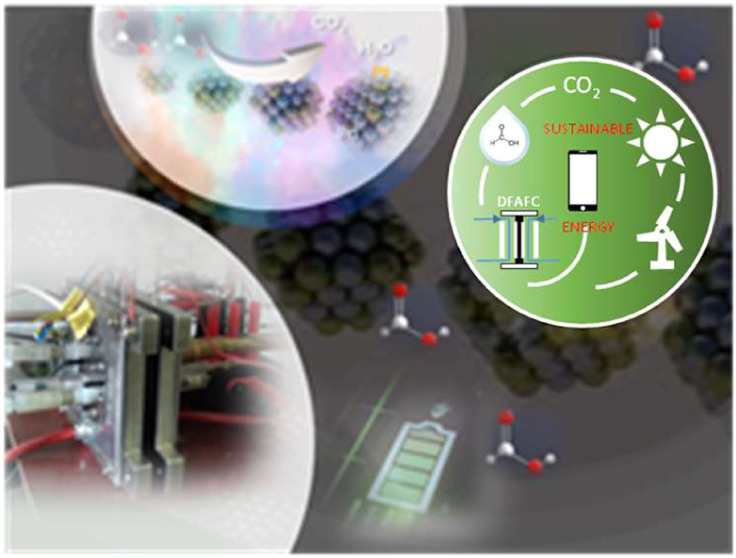

## 1 Introduction

Hydrogen-powered polymer exchange membrane fuel cells (PEMFs) are currently the most promising low-temperature direct fuel cells with a proton-conducting polymer electrolyte.^1^ They have the highest power density; however, a significant challenge to the widespread adoption of hydrogen fuel cells is the technical difficulty associated with hydrogen distribution and storage.^2^ In contrast, liquid fuels offer several advantages over compressed hydrogen, primarily due to their higher energy density per unit volume and the ability to be stored in lightweight plastic containers.^3^ Among liquid fuel cells, the direct formic acid fuel cell (DFAFC) has emerged as a promising option for powering portable electrical devices.^4^

The main advantages of the DFAFC include its high theoretical open-circuit voltage (1.45 V), high power density, and the use of a nearly nontoxic fuel.^5^ While formic acid (FA) has a lower energy density (1.63 Wh/kg) compared to methanol (6.07 Wh/kg), this limitation is mitigated by the significantly lower (several times) crossover of FA through the Nafion membrane to the cathode side.^6^ This reduced crossover is due to electrostatic anionic repulsion between formate ions (HCOO^–^) and sulfate ions (SO_3_^2–^) within the membrane.^6^ Studies have shown that the apparent diffusion coefficient of formic acid through Nafion is three times lower than that of methanol, which improves both fuel and voltage efficiency in DFAFCs. Furthermore, the voltage efficiency of DFAFCs is increased by the higher activity of the anode catalysts in the electrooxidation reaction of FA compared to methanol electrooxidation.^7^ Finally, the cost of FA production can be minimized through new technologies, such as the electroreduction of CO_2_ and biomass, offering a sustainable, economically viable, and environmentally friendly method for fuel production.^8^

Electrocatalytic oxidation of FA is most often described by two mechanisms, depending on the catalyst employed.^9^ In the direct pathway, HCOOH undergoes direct dehydrogenation, resulting in the production of CO_2_ mediated by one or more active intermediate compounds. In contrast, the indirect mechanism involves the dehydration of formic acid to form carbon monoxide (CO). This process is strongly dependent on the potential applied to the working electrode. The CO adsorbed on the surface of the catalyst can be further oxidized to CO_2_ or poison the catalyst.

Research is underway to develop an efficient catalyst for FA oxidation and bring DFAFC to the commercial market.^10^ Among them, bimetallic Pd–Pt-based nanoparticles have gained significant attention in DFAFCs due to their enhanced catalytic properties compared to single-metal catalysts.^11^ The Pd catalyst is known for its excellent direct FA oxidation activity, mainly due to its much higher ability to prevent CO poisoning compared to the Pt catalyst.^12^ Pd tends to break only the O–H bonds in the HCOOH molecule in the entire potential range, and therefore, almost exclusively, the direct oxidation of FA to CO_2_ occurs on it, so that during rapid tests of initial activity, the degree of carbon monoxide coverage is close to zero.^13^ Pt, on the other hand, tends to break C–O and/or C–H bonds at low overpotential, and O–H bonds at high overpotential;^13^ as a consequence, FAO proceeds on Pt mainly via indirect dehydration pathways. For this, the initial rate of the FAO reaction is much higher on Pd than on Pt, on which a part of the surface is already covered with CO_ad_ during the determination of the initial activity. Therefore, bimetallic Pd–Pt alloys effectively reduce reaction intermediates and increase both overall catalytic performance and catalyst stability, which is further enhanced by the use of conductive nanostructured support materials with highly developed surface areas.^14^ Nevertheless, the degradation of Pt and Pd catalysts during long-term electrochemical testing is one of the main weaknesses of these materials. This results in a progressively decreasing catalytic activity and, therefore, performance of the entire fuel cell. Reproducible and low-cost preparation of Pt–Pd nanoparticles of similar size and their incorporation into a scaffold of well-developed conductive nanomaterials, enabling maximum utilization of active centers with long-term stability on the DFAFC anode, are the main challenges in the synthesis of these catalysts.

In addition to Pd–Pt, Pd alloys with other metals such as Cu,^15^ Ag,^16,17^ Au,^17^ Sn,^18^ Ni,^19^ Rh,^20^ Pb,^21^ Ce,^22^ In,^23^ Bi,^24^ Cd,^24^ Cu, and Co^25^ showed an increase not only in the catalytic activity but also improved Pd’s corrosion resistance.

Among the various methods of synthesizing bimetallic alloys are electrodeposition, coprecipitation, and hydrothermal methods.

Pd–Pt NPs offer a synergistic effect that improves the efficiency and stability of the fuel cell. The bimetallic Pd–Pt NPs enhance the electrochemical properties by optimizing the electronic structure and increasing the number of active sites for FA oxidation.^26^ The unique interaction between Pd and Pt can reduce the poisoning effect caused by intermediate species, such as CO, which commonly hinders the performance of pure Pt catalysts. This results in better long-term stability and higher catalytic activity at lower overpotentials.^27^ Furthermore, Pd–Pt bimetallic NPs exhibit a high surface-to-volume ratio, which is advantageous for maximizing the catalytic efficiency in DFAFCs.^28^ These NPs can also be tailored in size and composition, providing further opportunities to fine-tune their properties for specific applications.^29–31^ Moreover, Pd–Pt NPs also exhibit high stability in acidic environments due to their chemically inert properties. This makes Pd–Pt bimetallic NPs promising materials for advancing the development of efficient, stable, and cost-effective DFAFCs.

The synergistic contribution of the two catalysts is exploited by fabricating various Pd–Pt bimetallic systems in the form of materials with different nanostructured properties.^12,32^ Various methods, including electrochemical deposition and chemical reduction, have been employed to fabricate Pt–Pd catalysts, often in the form of NPs or nanostructured materials to increase the surface area and improve the electrochemical properties.^29,33^ Research has shown that different morphologies, such as flower-like or leaf-like dendritic structures, can be formed depending on the deposition potential, which affects the catalyst’s performance in fuel cell polarization studies.^34^ In another study, the Pd_*x*_Pt_*y*_/TiO_2_ catalyst was prepared by reducing colloidal metal nanoparticles of mixed K_2_PdCl_4_ and K_2_PtCl_6_, Pd and Pt precursors, respectively, with poly­(vinyl)­alcohol (PVA) as a stabilizing agent to control the nanoparticle size.^35^ Pt–Pd-based catalysts have demonstrated improved power densities and efficiency compared to pure Pt or Pd, with studies showing promising results in terms of both catalytic activity and long-term stability in DFAFCs.

Recently, the electrochemical deposition of metal catalysts has attracted increasing attention due to its advantages, including the high purity of deposits, simple production methods, and the ability to precisely control the catalyst loading. Pt–Pd bimetallic catalysts were codeposited under cyclic voltammetry conditions on a carbon black-coated carbon paper.^36^ Depending on the potential ranges, flower-like or leaf-like dendrites were formed at 0–1.3 V or −0.2 to 1.3 V vs SHE, respectively. As Pt precursors, H_2_PtCl_6_ or K_2_PtCl_4_ were utilized. The leaf-like structures performed better in the fuel cell polarization studies at 70 °C, obtaining a maximum power density of 49 mW cm^–2^. In comparison, flower-like structures showed a power density of 20 mW cm^–2^.

In this research work, the Pd_0.64_Pt_0.36_ (0.64:0.36 at. ratio in the metal phase) and Pd_0.50_Pt_0.50_ (0.50:0.50 at. ratio in the metal phase) catalysts were electrochemically prepared under potentiodynamic conditions by using linear sweep voltammetry (LSV). Pd–Pt NPs were formed by electroreduction of PtCl_4_ and Pd­(OAc)_2_ precursors, previously immobilized inside the MWCNTs network deposited on a carbon cloth. Our deposition procedure resulted in spherical nanoparticles, ∼4 nm in diameter, which are well-separated from each other and uniformly decorate the entire MWCNT surface. XRD analysis showed the presence of Pd- and Pt-rich phases, while DRIFT measurements clearly indicate that the catalyst is much more resistant to CO poisoning compared with monometallic Pd and Pt catalysts. The Pd_0.64_Pt_0.36_ catalyst has a high ECSA value (56.94 m^2^/g) and above 80% active site availability. These parameters explain its high activity and stability toward FA electrooxidation. The performance of the Pd_0.64_Pt_0.36_ and Pd_0.50_Pt_0.50_ catalysts as an anode was evaluated in a DFAFC cell under a 50 mA/cm^2^ load, a temperature of 60 °C, and an airflow of 200 mL/min. A long-term stability study measured (14 h) for 3 M HCOOH showed excellent durability of the catalyst. DFAFC with the Pd_0.64_Pt_0.36_ catalyst shows an excellent power density value of 64 mW/cm^2^ at 250 mA/cm^2^ recorded at 60 °C for airflow on the cathode on the catalyst previously stabilized in the cell operating conditions. In order to reduce the fuel cell costs, testing was carried out at low metal loads on the anode (0.6 mg of Pt_*x*_Pt_1–*x*_), which is several times lower than those typically used in DFAFC.

## 2 Experimental Section

### 2.1 Chemicals

Multiwalled carbon nanotubes (MWCNTs) of >95% purity, ∼20 nm diameter, and 1–25 μm length were from CNT Co., Ltd. (Korea). Palladium­(II)­acetate (47% Pd; Pd­(OAc)_2_) and platinum­(IV) chloride (99.99%; PtCl_4_) were purchased from Sigma-Aldrich and used without further purification. 5% Nafion solution was from DuPont Fluoroproducts. Carbon cloth was obtained from the Fuel Cell Store. All solutions were prepared by using deionized (Millipore Milli-Q) water. Argon (BIP plus, Air Products) was used to deaerate the solutions for electrochemical experiments.

### 2.2 Preparation of Functionalized MWCNTs

MWCNTs were functionalized with concentrated nitric acid (V) by annealing for 8 h at ∼120 °C under a reflux condenser. After functionalization, the MWCNTs were filtered and washed with distilled water until the filtrate reached a pH of 6.5 and then dried on a heating plate. An appropriate amount of distilled water was added to obtain a suspension with a weight concentration of 0.4%. The suspension was then dispersed in an ultrasonic bath and further homogenized through three cycles in the homogenization chamber of a Microfluidics M-110P high-pressure homogenizer at 200 bar.

### 2.3 Catalytic Ink Preparation

2.7 g portion of 0.4% CNTs suspension in H_2_O was mixed with 72 mg of 5% Nafion solution, 2.78 mg of PtCl_4_, and 2.93 mg of Pd­(OAc)_2_. Pd­(OAc)_2_ is poorly soluble in H_2_O; therefore, it was first dissolved in 120 μL of acetonitrile. The vial with the mixture was then placed in an ultrasonic bath (Elma, Transsonic Digitals) for 1 h to mix all of the components and obtain a homogeneous ink.

### 2.4 Pt–Pd Catalyst Preparation and Electrochemical Characterization

Catalytic ink was pipetted in portions onto the 5 cm^2^ surface of a carbon cloth layer by layer. Note, that successive portions of ink were deposited and spread as thin layers after the solvent from the previous layer was thoroughly evaporated. The deposition was completed when a total mass of catalytic material of ∼4 mg/cm^2^ was obtained (Figure S1). The electrodes thus prepared contained 0.6 mg/cm^2^ Pd/Pt, in the form of Pd/Pt precursors, with a Pd/Pt weight ratio of 0.9. This corresponds to the following molar fractions of metals in the metallic phase: *x*_Pd_ = 0.61 and *x*_Pt_ = 0.39.

Pd and Pt nanoparticles were formed by the electroreduction of PtCl_4_ and Pd­(OAc)_2_ precursors immobilized inside the MWCNTs network. Measurements were performed for 0.5 M H_2_SO_4_ under potentiodynamic conditions by using linear sweep voltammetry (LSV). For this purpose, an electrochemical cell was constructed in which the working electrode (WE) with an area of 5 cm^2^ was a carbon cloth with deposited catalytic material. A Pt wire and a Ag/AgCl electrode in 3 M KCl (Mineral, Poland) were used as the auxiliary and reference electrodes, respectively. The system was enclosed in an environmental chamber, providing an inert gas atmosphere. The same cell was utilized for all of the electrochemical measurements conducted with a BioLogic VSP potentiostat/galvanostat (BioLogic Sciences Instruments) at a controlled room temperature of ∼21 °C. Before each measurement, the electrolyte was saturated with Ar.

LSV deposition was carried out in the potential range of 0.6 to −0.2 V at a scan rate of 10 mV/s. During the measurements, argon flowed over the solution’s surface, ensuring anaerobic conditions. A charge of ∼4 C (calculated from the area under the curves *i* = *f*(*t*) recorded during electroreduction of PtCl_4_ and Pd­(OAc)_2_) passed through the system corresponding to Pd and Pt nanoparticles deposition on the electrode surface with masses of 1.39 and 1.61 mg, respectively. This value is consistent with the nominal charge density of metals resulting from the preparation methodology. The Pd and Pt loadings are, respectively, 0.278 and 0.322 mg/cm^2^. The resulting anodic catalyst has atomic ratios in the metallic phase of *x*_Pd_ = 0.64 and *x*_Pt_ = 0.36, and metal weight percent (wt%) in the metallic phase equal to wt%_Pd_ = 45.9% and wt%_Pt_ = 54.1%. After electrodeposition, the anode was rinsed with deionized water and dried at 60 ^◦^C in a vacuum oven.

CO-stripping experiments were carried out in a 0.5 M H_2_SO_4_ solution. First, a 0.5 M H_2_SO_4_ solution was purged by Ar for 30 min. Then, CO was purged into the solution for 90 min, keeping the potential at 0.2 V vs Ag/AgCl, i.e., in the double layer potential range to avoid simultaneous hydrogen absorption in the electrode bulk.^37^ After removing the dissolved CO gas by purging Ar for 30 min at an electrode potential of 0.2 V vs Ag/AgCl, CO stripping was performed in the potential range of −0.2 to 1.3 V vs Ag/AgCl. The electrochemically active surface area (*ECSA*) of the catalyst was determined both from the surface oxide formation region, assuming 424 μC/cm^2^ for monolayer adsorption of Pt and Pd oxides on the catalyst surface, and using CO-stripping voltammetry, assuming 420 μC/cm^2^ for monolayer adsorption of CO on the catalyst surface.^38^

### 2.5 Test Procedure for Formic Acid Electrooxidation over Pd_0.64_Pt_0.36_ Catalysts in DFAFC

The test was performed in a single fuel cell made of two stainless steel holders, two graphite plates with serpentine-shaped grooves covering an area of 5 cm^2^ each, a tested anode, a cathode, and a pretreated Nafion N115 membrane.

The Nafion N115 membrane pretreatment consisted of soaking it in 3% H_2_O_2_ for 1 h at 80 °C, then in deionized water for 2 h at 80 °C, and finally in 0.5 M H_2_SO_4_ for 1 h at 80 °C. After each step, the membrane was thoroughly rinsed with deionized water to remove any residue.

A commercial 60% Pt/Vulcan XC-72 Premetek catalyst was used to prepare the cathode. First, the ink was prepared. To 46.8 mg of catalyst were added 1.93 mL of water, 0.28 mL of isopropyl alcohol, and 401 mg of 5% Nafion dispersion. The ink mixture was sonicated for 30 min to obtain a uniform dispersion. The ink was applied to 30% Wet Proofed Carbon Cloth (FuelCellStore) using an airbrush until a loading of 4 mg_Pt_/cm^2^ was achieved. Finally, the electrode was dried at 130 °C for 30 min.

The graphite plates, electrodes, and a membrane were compressed between holders at a pressure of 10 bar to provide good electrical and ionic conductivity (Figure S2).

The measurement procedure using the fuel cell began by heating it to 60 °C and supplying water at a flow rate of 1.5 mL/min and oxygen at a flow rate of 50 mL/min to the anode and cathode, respectively. Then, the water was changed to 3 M FA solution, and an activity test was performed. After the test was completed, the FA was changed back to water, and a regeneration process was carried out by letting the water run on the anode until the cell voltage dropped to below 100 mV and stabilized. Next, an anode activity test was carried out by applying hydrogen to the cathode instead of oxygen at a rate of 20 mL/min. After the test was completed, hydrogen was changed back to oxygen to regenerate the catalyst. The cathode was then supplied with air at a flow rate of 200 mL/min, and formic acid was applied to the anode to conduct a stability test for 14 h at a current density of 50 mA/cm^2^. This measurement was then continued by periodically increasing the current density to determine the maximum power of the cell for each catalyst.

### 2.6 Physical Characterization

The prepared Pd_0.64_Pt_0.36_ catalyst was characterized for particle morphology using the field-emission scanning electron microscope (FE-SEM) FEI Nova NanoSEM 450 Series under high vacuum (∼10^–6^ mbar). The samples were fixed with a conductive carbon tape to the typical SEM specimen stub for precise positioning under the electron column for imaging. SEM images were collected using the Through Lens Detector (TLD) of secondary electrons at a primary beam energy of 10 keV and a 4.8 mm working distance from the pole piece. All images were obtained at a long scan acquisition time (20 μs) of typically 30 s per image after choosing the inspection region. EDS measurements were performed using an EDAX Octane Elect system equipped with a silicon drift detector (SDD) technology. All measurements were conducted under the same conditions as SEM imaging but with a higher electron beam energy of 15 keV. Data was acquired from a selected region, typically 2 × 2 μm^2^ in size.

X-ray diffraction (XRD) measurements were performed using a Bruker D5000 X-ray diffractometer with a copper-sealed tube (40 kV, 40 mA), and a LynxEye detector in the Bragg–Brentano configuration. All scans were performed at room temperature in the range of scattering angle 2Θ from 10 to 100° with a step of 0.02° and a counting time of 1s/step. The sample was mounted in a glass holder. To check the sample phase homogeneity, the scans were performed in a flow of a mixture of hydrogen and helium (ratio 1:10). XRD patterns were analyzed with FITYK.^39^ The Scherrer equation was used to estimate the average size of the crystallites from the calculated integral peak width. All calculations were made assuming a Cu Kα wavelength equal to 1.5418 Å.

High-resolution transmission electron microscopy (HR-TEM) and scanning transmission electron microscopy (STEM) in high-angle annular dark-field (HAADF) mode for elemental mapping were performed using an FEI TITAN G2 60–300 microscope with an X-FEG-type emission gun operating at 80 kV. Images were taken with an UltraScan 1000XP CCD camera (Gatan). Energy Dispersive Spectrometry (EDS) was acquired in Scanning TEM (STEM) mode by the Super-X system with four silicon drift detectors (Bruker). STEM images were taken with the HAADF detector 3000 (Fishione). The powder samples were dispersed in ethanol and sonicated for 5 min. One drop of the resulting solution was then placed on a copper grid with a holey carbon film, after which the sample was dried at room temperature.

The chemical composition of the Pd_0.64_Pt_0.36_ catalyst was measured by XPS spectroscopy using a Microlab 350 instrument (Thermo Electron). XPS spectra were acquired using AlKα (hν = 1486.6 eV) radiation. Survey and high-resolution spectra were recorded using pass energies of 100 and 40 eV, and the XPS signal intensity was determined using Smart background subtraction. Peaks were fitted using an asymmetric Gaussian/Lorentzian mixed function, and the measured binding energies were corrected based on the C 1s energy at 284.8 eV.^40^

*Operando* DRIFT (diffuse reflectance infrared Fourier Transform spectroscopy) studies were performed in a continuous flow Harrick Praying Manti reaction chamber. An FTIR Nicolet Nexus 470 spectrometer was used to study the catalyst’s surface performance in situ during the CO adsorption–desorption experiments. The reactions were carried out at a temperature range of RT–343 K. The temperature was adjusted and controlled by a Thermo Scientific voltage controller with a K-type thermocouple. Spectra were recorded in diffuse reflectance mode, and 45 scans were collected at a resolution of 1 cm^–1^.

Temperature-programmed CO adsorption–desorption experiments (CO experiments) were carried out over the prereduced catalyst. The reduction was carried out at 343 K in 5% H_2_ in He for 120 min. For all experiments, first, CO was admitted to the reactor at RT, and at the CO saturation point, the reactor was purged by He, and then the catalyst sample was heated upon He flow up to 343 K with a ramp of 10 K/min. The gas flow was kept constant at 100 mL min^–1^.

## 3 Results

### 3.1 Electrode Material Characterization

Figures 1a,b and S3a show transmission electron microscopy (TEM) and scanning electron microscopy (SEM) images, respectively, of MWCNTs decorated with Pd_0.64_Pt_0.36_ NPs. The NPs are mainly spherical, but oblate and elliptical objects are also visible. They are separated from each other, evenly decorating the MWCNT’s surfaces. Our procedure, which involves the potentiodynamic deposition of Pd–Pt nanoparticles from PtCl_4_ and Pd­(OAc)_2_ precursors, previously entrapped in the MWCNTs network, leads to homogeneous particle formation with good dispersion on the MWCNTs/carbon cloth support.

**Figure 1:**
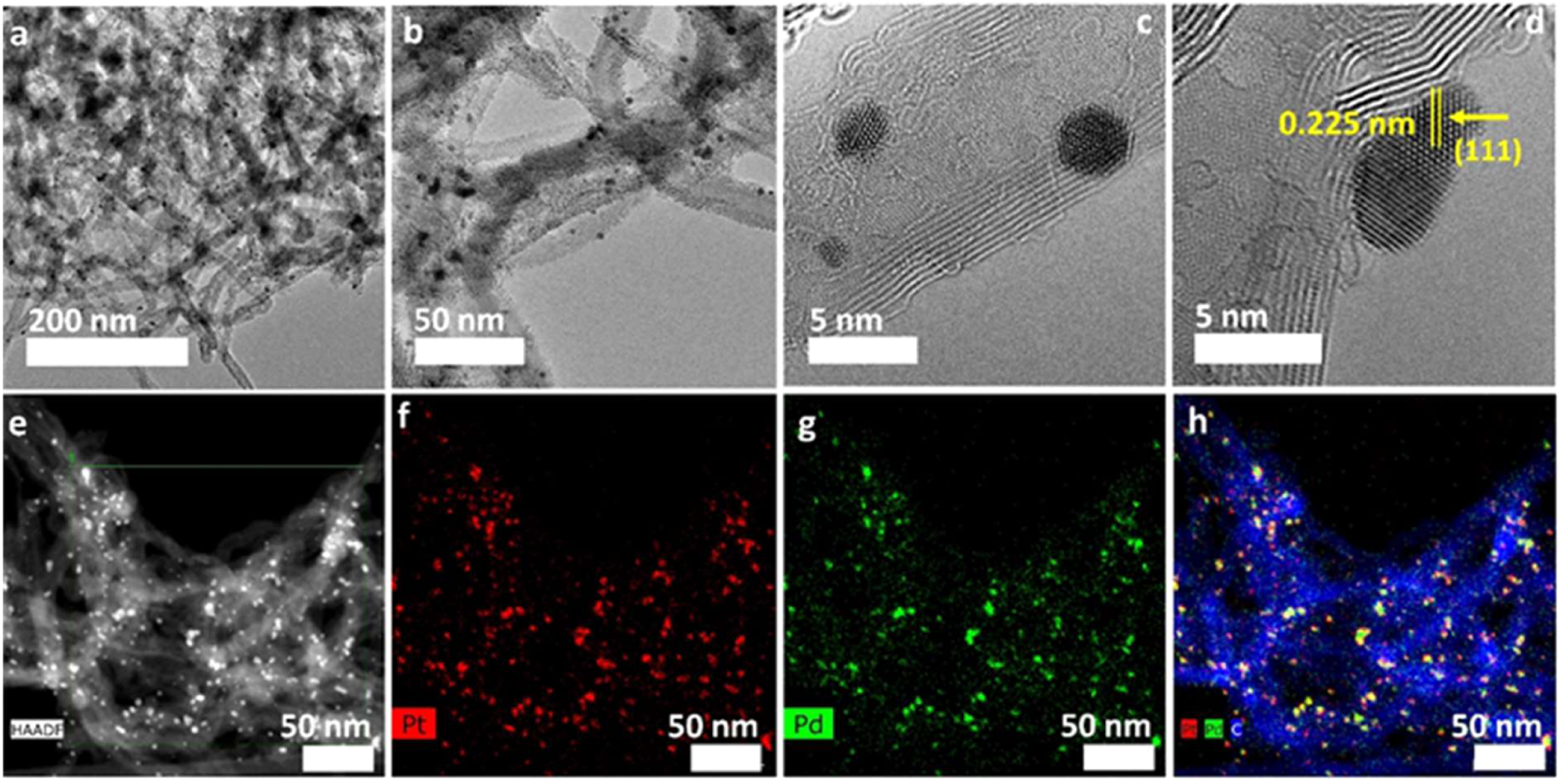
(a, b) TEM, (c, d) HRTEM, and (e) HAADF-STEM images of the Pd_0.64_Pt_0.36_ catalyst. (f–h) Elemental mapping of (f) platinum, (g) palladium, and (h) platinum, palladium, and carbon together for the Pd_0.64_Pt_0.36_ catalyst.

Figure 1c shows a high-resolution TEM (HRTEM) image of an individual, most likely an icosahedron or cuboctahedron nanoparticle with an edge length of ∼2.3 nm and a diagonal of ∼4.7 nm. Similar NPs were obtained for the silsesquioxane-stabilized Pt_0.7_Pd_0.3_ alloy.^31^ Moreover, the core–shelled Pt@Pd catalyst of icosahedron shape revealed higher activity for FA oxidation than that of the cube or dodecahedron shape.^32^ The fringes in the nanoparticles showing a period of 0.225 Å can be indexed as (111) facets, as expected for a face-centered cubic (fcc) lattice (Figure 1d). It was found that Pt NPs with a preferential orientation of (111) are most active toward FA oxidation due to the adsorption strength of intermediate compounds.^41^ High-angle annular dark-field scanning transmission electron microscopy (HAADF-STEM) (Figure 1e) and corresponding elemental mapping images (Figure 1f,g) confirmed the Pd and Pt presence well-dispersed over the MWCNTs network.

Structural characteristics of Pd_0.64_Pt_0.36_ NPS were confirmed by the XRD patterns. Figure 2a shows the XRD patterns of the Pd_0.64_Pt_0.36_/MWCNTs/carbon cloth measured in air. The characteristic diffraction patterns (111), (200), (220), (113), and (222) of the *fcc* structure can be seen, indicating the crystalline nature of the nanoparticles according to the HRTEM results. Additional peaks, marked with stars, are associated with the carbon phases.

**Figure 2:**
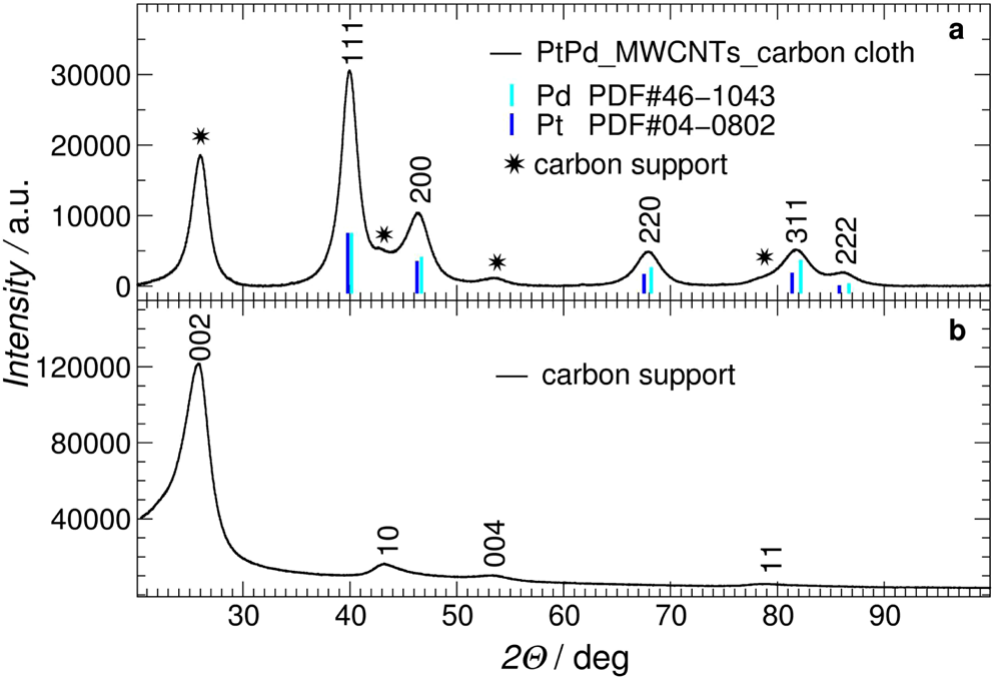
XRD patterns of (a) Pd_0.64_Pt_0.36_/MWCNTs/carbon cloth catalyst and (b) bare MWCNTs/carbon cloth under air. The peaks marked with an asterisk in Figure 2a correspond to the peaks from the carbon phase shown in Figure 2b.

Figure 2b shows the XRD profile for carbon phases only, i.e., bare MWCNTs deposited onto carbon clothes in the Pt–Pd nanoparticle’s absence. The carbons are easily penetrable by X-rays, so the patterns also include an amorphous contribution from the carbon cloth and glass holder. The MWCNTs/carbon cloth sample shows the diffraction peaks at 2Θ ∼25.5°, ∼43°, ∼54°, and ∼78°, which are indexed to the (002), (10), (004), and (11) plane/band, respectively, which correspond very well to signals marked with stars for the Pd_0.64_Pt_0.36_/MWCNTs/carbon cloth (Figure 2a).

Figure 3a shows the XRD profile of the Pd_0.64_Pt_0.36_ catalyst measured under an air atmosphere. The left-side asymmetry of the 111 profile is apparent, and the intensity of this profile is higher than expected for the *fcc* structure compared with the 200 and 220 profiles.

**Figure 3:**
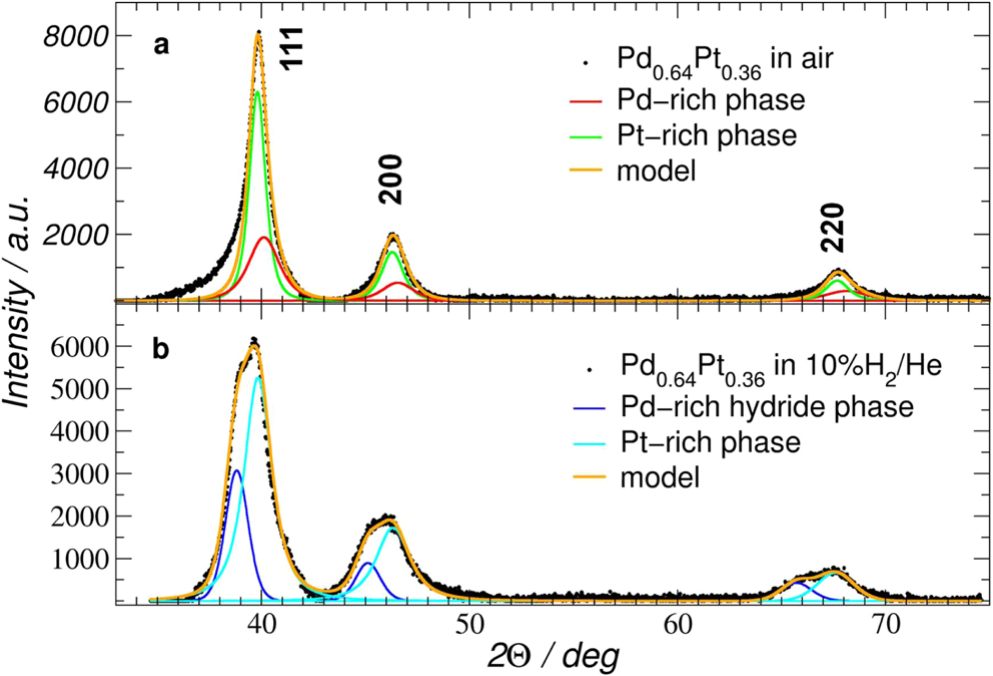
XRD patterns of Pd_0.64_Pt_0.36_ under (a) air and (b) H_2_ atmosphere after background removal, normalization, and angle 2Θ correction. Black dots mark experimental points. For each graph, the sum of the model functions of the two phases is shown as an orange line.

Usually, the diffraction patterns of nanocrystals differ from those of polycrystalline solids. For example, for the nanocrystal material, the amplitude ratio of 200 and 111 reflexes is smaller than that for polycrystalline solids. Moreover, the distance between the center positions of these reflexes gets smaller and the positions of the center of reflexes do not strictly obey Bragg’s law following right- or left-sided asymmetry of the profile.^42^ For example, the left-side asymmetry of 111 can be caused by a slightly expanded surface component of the nanocrystals. Figure 3b shows the XRD profile of the Pd_0.64_Pt_0.36_ catalyst measured under a hydrogen atmosphere. As can be seen, each profile splits into two. This points out that the metallic phase contains both a Pd-rich (undergoing a transition to β hydride) and a Pt-rich phase (not affected by H_2_). Therefore, model functions corresponding to the Pd-rich and Pt-rich phases, respectively, were fitted to each profile. The lattice parameters were calculated and listed in Table S2. Using Scherrer’s equations, the average crystallite size was estimated to be 6 and 5 nm for the Pd- and Pt-rich phases, respectively, which very well corresponds to the size estimated from the HRTEM imaging.

XPS measurements provided details about the chemical composition of the surface of the Pd_0.64_Pt_0.36_/MWCNTs catalyst. For this catalyst, the peaks assigned to C, O, F, Pd, and Pt atoms are distinguished (Figure S4). The spectrum within the binding energy (EB) range of the Pd 3d electrons shows three pairs of doublets, indicating the presence of three different forms of palladium (Figure 4a). One pair of the most intense peaks, located at 335.6 and 340.9 eV, corresponds to the metallic Pd^0^.^43^ The pair of peaks located at 336.5, 341.9 eV, and 338.0, 343.0 eV are attributed to Pd^2+^ and Pd^4+^, respectively.^44^ Similarly, we observed for Pt, where both metallic and oxidized forms occur at 71.2, 74.4 eV, and 72.7, 75.8 eV, respectively (Figure 4b).^44^ The mole ratio of Pd/Pt ≈ 1.63 was determined from the relative integrated intensities of the respective Pd 3d and Pt 4f core-level spectra. This value is close to 1.78 found from the synthesis method of the Pd_0.64_Pt_0.36_/MWCNTs/carbon cloth.

**Figure 4:**
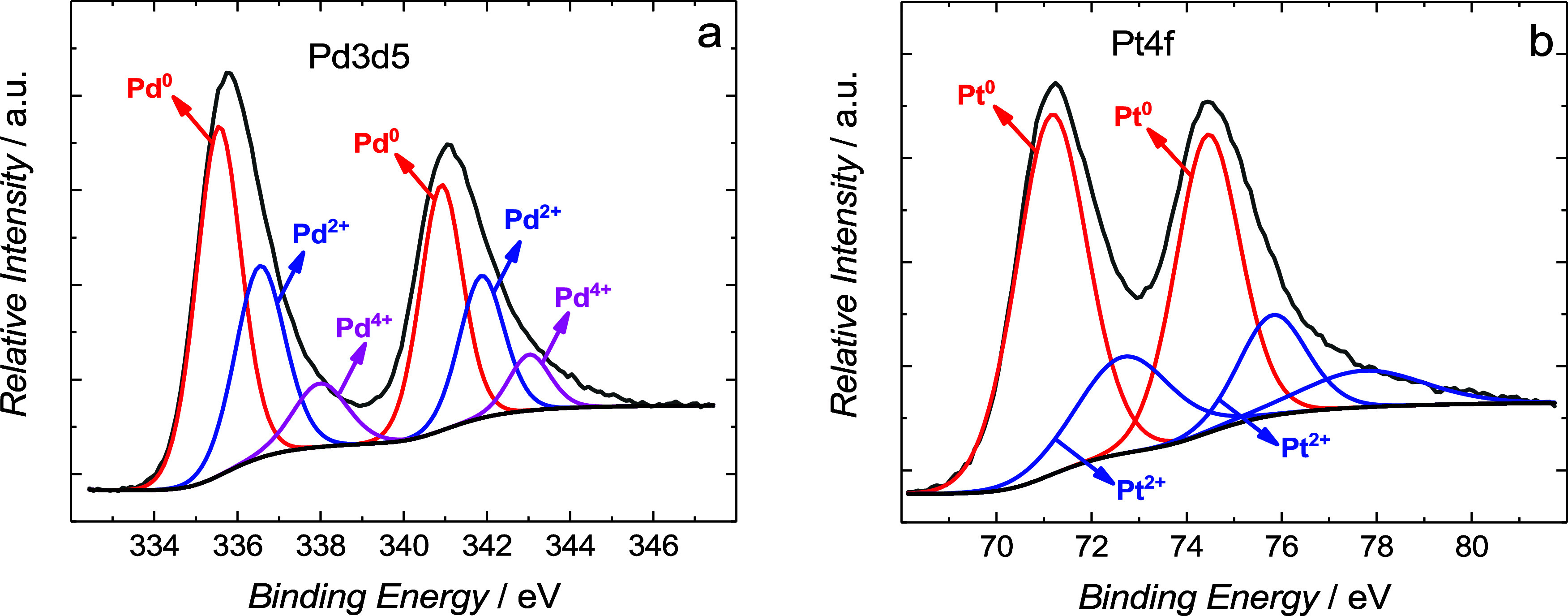
X-ray photoelectron spectroscopy spectra of (a) Pd 3d and (b) Pt 4f for the Pt–Pd NPs supported on MWCNTs.

Based on the XPS measurement, the metallic composition of the Pd_0.64_Pt_0.36_ catalyst is shown in Table S3. The Pd-to-Pt atomic ratio of the studied catalyst, calculated from XPS spectra, is Pd_0.62_Pt_0.38_, which is similar to that calculated from the precursor metal and electrodynamic deposition. In addition, the catalyst composition was confirmed by EDS studies, which showed that the Pd-to-Pt atomic ratio is Pd_0.71_Pt_0.29_ (Figure S3b and Table S1). The slight discrepancies may be due to the surface nature of XPS measurements, while EDS is a volumetric technique.

The *operando* DRIFT results are shown in Figure 5. CO as an IR probe molecule shows a high sensitivity of the carbonyl stretching frequency ν­(CO), which is influenced by the oxidation and coordination states of Pd and Pt cations. The use of CO as a probe molecule for characterizing the structural properties and charge states of supported Pd and Pt catalysts has been well-established in the literature. Figure 5A shows the DRIFT results for the Pd reference catalyst. For supported Pd^0^ particles (Pd^0^–CO), the vibrational frequency of adsorbed CO is usually observed at ∼2100 cm^–1^.^45^ In contrast, when CO is adsorbed to oxide-supported cationic Pd species (Pd^3+^-CO, Pd^2+^-CO, Pd^+^-CO), a blue shift in the CO vibrational frequency is noted, with values exceeding 2100 cm^–1^, especially Pd^3+^–CO (2215–2195 cm^–1^), Pd^2+^–CO (2215–2140 cm^–1^), and Pd^+^–CO (2145–2100 cm^–1^).^46^ However, the spectrum from Figure 5A showed almost no intensity in the frequency range associated with CO adsorbed to cationic Pd sites (>2100 cm^–1^), suggesting the presence of fully reduced Pd clusters.

**Figure 5:**
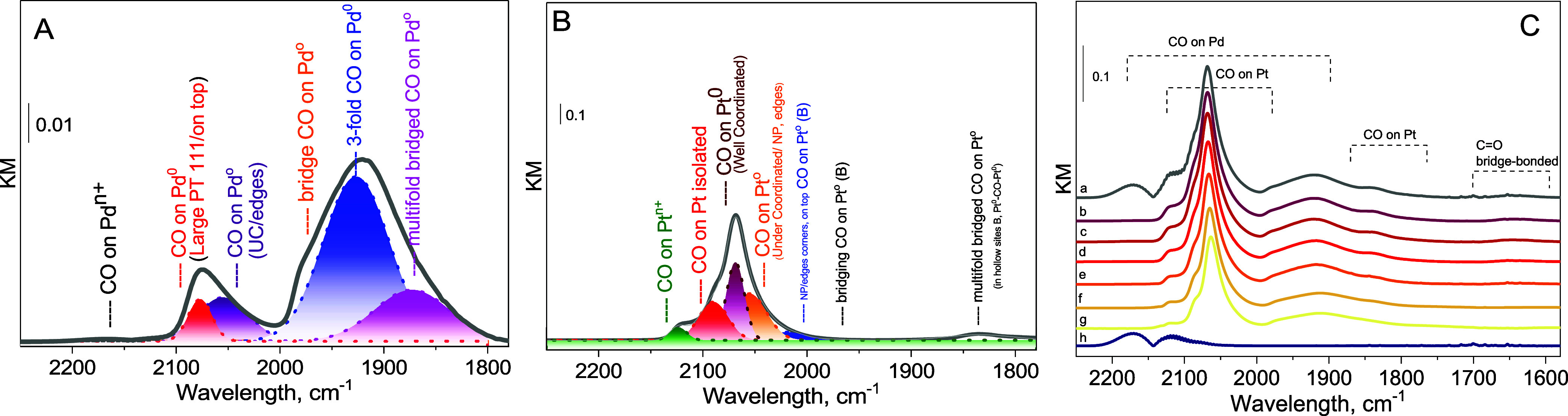
*Operando* DRIFT spectra of CO adsorbed on prereduced samples of (A) Pd and (B) Pt reference catalysts and (C) Pd_0.64_Pt_0.36_ NPs supported on MWCNTs; (a–h) spectra recorded for Pd_0.64_Pt_0.36_ upon heating up to 343 K with the ramp of 10 K/min and He purge.

Similarly, for the Pt reference catalyst shown in Figure 5B, an almost complete reduction of the active phase was observed, as indicated by the absence of intensity in the frequency range associated with CO adsorbed on Pt cationic sites (>2100 cm^–1^). The spectrum in Figure 5B shows two predominant CO stretching bands centered at 2077 cm^–1^, which overlap in the region associated with CO adsorbed on Pt^0^ sites, assigned to well-coordinated (WC) Pt sites, and at 2058 cm^–1^ assigned to under-coordinated (UC) Pt sites.^47^ For supported Pt particles when CO is adsorbed to Pt^0^ sites, the vibrational frequency increases from 2040 to 2100 cm^–1^ as the Pt–Pt coordination number increases from ∼5 (characteristic of corners or defect sites on Pt particles) to 9 (characteristic of extended (111) surfaces on Pt particles). When CO is adsorbed to oxide-supported cationic Pt species (Pt_ox_ clusters, Pt_iso_ species, or Pt coordinated with oxidizing ligands), the vibrational frequency of CO is blue-shifted, typically exceeding 2100 cm^–1^. The blue shift in the CO vibrational frequency for cationic Pt sites compared with Pt^0^ sites arises from reduced charge transfer to CO and a CO bond distance that is closer to the gas-phase CO distance. For the in situ pretreated catalyst (1 h at 523 K in H_2_), any remaining IR bands of CO adsorbed to Pt sites with stretching frequencies >2100 cm^–1^ due to the presence of any Pt oxidized clusters have vanished.

Figure 5C *line a* shows the IR spectra of CO adsorbed at saturation coverage (*line a*) and room temperature (*line b*) over the Pd_0.64_Pt_0.36_ catalyst (*line h* corresponds to the pretreated catalyst). The *operando* DRIFT spectra obtained for the bimetallic catalyst showed the complexity of the adsorption peaks in the CO and carbonates region, which is attributed to surface heterogeneity and the presence of both the Pd and Pt components. The signal observed at 2060 cm^–1^ corresponds to the C–O internal stretching vibration mode of the CO adsorbed on-top Pt (111).^47^ The intensity of the CO stretching mode for linear CO at ∼2060 cm^–1^ remains constant upon heating from room temperature (Figure 5C, *line b*) to 343 K (Figure 5C, *line g*). The shoulder at ∼2115 cm^–1^ can be ascribed to the adsorption of CO on both isolated Pd or Pt sites or, less likely, to a direct adsorption on the MWCNTs surface.^48^ The additional signals observed at 1698 and 1716 cm^–1^ for the prereduced Pd_0.64_Pt_0.36_ most likely correspond to a bridge-bonded configuration with C=O, although a definitive assignment has not yet been made.^49,50^ The data indicate that the Pd_0.64_Pt_0.36_ catalyst is able to efficiently store CO up to 343 K and to remove CO from the surface upon heating. Moreover, upon temperature increase, the multifold CO bridged on Pd and Pt sites diminishes first, suggesting a stronger CO bonding for on-top Pt (111) than Pd or Pd_*x*_Pt_*y*_ domains.

CO-probe molecule infrared (IR) spectroscopy is a valuable characterization technique that allows the probing of the local structure, oxidation state, and coordination environment of supported precious metals. The technique is also site-specific due to the varying vibrational frequency of CO when adsorbed to different types of supported metal sites, and it can be operated in a temperature-programmed manner to extract information about the chemical reactivity of distinct precious metal sites.^51^

Isolated and oxidized clusters require consideration, as previous reports on these species identify their stretching frequencies, both existing in the range of ∼2080 to 2130 cm^–1^. A similar vibrational frequency of CO when adsorbed to Pt_iso_ and Pt_ox_, which is blue-shifted (higher frequency) from the stretching frequency of CO on Pt^0^ (2030–2100 cm^–1^), arises from the similar cationic charge of Pt in both structures.^52^ The importance of distinguishing these species is underscored by reports showing that Pt_ox_ species—ranging from small clusters containing a few Pt atoms to extended single-crystal surfaces—interact strongly with CO and exhibit minimal CO oxidation reactivity. This interaction could obscure measurements of the Pt_iso_ reactivity if these species are not differentiated and distinctly compared. The previous study revealed that the interaction strength between CO and precious metals follows the order: Pt_iso_ ≪ Pd_iso_ < Pd^0^ < Pd_ox_ < Pt^0^ < Pt_ox_. Moreover, it was found that for steady-state CO oxidation measurements (free from heat and mass transfer effects), Pt_iso_ exhibited a 2-fold higher turnover frequency (TOF) than 1 nm Pt^0^ clusters at 473 K, although both species followed an identical reaction mechanism.^53^

These results suggest that the active site for CO oxidation on supported Pt is composed of interfacial cationic Pt atoms. Consequently, when supported, Pt_iso_ species exhibit optimal reactivity on a per mass and site basis as every atom is exposed and accessible for a given reaction. In our study, insights into the potential synergistic effects in Pt–Pd systems have been provided. The findings from the DRFT study suggest that the Pt–Pd bimetallic NPs exhibit a synergistic effect that enhances the antipoisoning properties, stability, and number of active sites, specifically as given below.

(i) *Poisoning resistance*: The results suggest a key aspect of synergy between Pt and Pd in terms of CO adsorption behavior. The weaker CO binding to Pd sites reduces the risk of CO poisoning, enhancing the catalyst’s antipoisoning capabilities (while considering the indirect mechanism of FA oxidation).^54^ Moreover, Pd could act by preventing CO from adsorbing too strongly on Pt and allowing for more efficient FA oxidation. FA adsorption (and probably simultaneous C–H bond activation) is the rate-determining step (RDS) for the direct pathway of FA oxidation.^54^ It has generally been suggested that this reaction occurs through weakly adsorbed reaction intermediates; however, the precise mechanism of the direct pathway and the characteristics of the weakly adsorbed intermediates remain subjects of ongoing debate. The *operando* DRIFT spectra indicate that CO adsorbs more strongly on the on-top Pt (111) surface than on Pd or mixed Pd*_x_* Pt*_y_* domains. This synergy could therefore improve the antipoisoning ability of the catalyst, leaving the surface accessible for carrying out the FA oxidation reaction.
(ii) *Stability*: The bimetallic catalyst shows stable behavior over a range of temperatures, indicating good thermal stability. The stability of the catalyst can be inferred from the fact that the DRIFT spectra for both the Pd and Pt reference catalysts (Figure 5A,B) show minimal intensity at frequencies associated with adsorbed CO on cationic sites (Pd^3+^, Pd^2+^, Pt^0^, and Pt_iso_). This suggests that the active phases in these catalysts are relatively stable during the experiment. In the case of the bimetallic Pd_0.64_Pt_0.36_ catalyst (Figure 5C), the *operando* DRIFT spectra show that CO is efficiently stored and removed up to 343 K, with CO bridges on Pd and Pt sites diminishing as the temperature increases. This behavior reflects good thermal stability and indicates that the catalyst can withstand moderate temperatures without significant deactivation or loss of active sites.

Furthermore, the interaction of CO with both Pd and Pt in the bimetallic system implies that the overall catalyst structure is likely more stable than that of individual monometallic Pt or Pd catalysts. The metal–support interactions, especially in the case of the Pd_0.64_Pt_0.36_ catalyst, might also contribute to the stability by providing additional support to the metal particles, preventing sintering and ensuring a more stable dispersion of the active metal sites over time.

(iii) *Number of active sites*: The presence of both Pd and Pt stabilizes the number of active sites, improving the overall catalytic performance and efficiency. The DRIFT spectra for the Pd_0.64_Pt_0.36_ catalyst show complex adsorption peaks due to the presence of both Pd and Pt components, indicating that both metals contribute to CO adsorption and catalytic activity. The observed CO stretching band at 2060 cm^–1^ corresponds to CO adsorbed on the well-coordinated Pt (111) sites, while the shoulder at 2115 cm^–1^ might correspond to isolated Pd or Pt sites.

This suggests that, considering the indirect mechanism, both Pd and Pt sites are active in the bimetallic system, and the presence of both metals likely stabilizes the number of accessible active sites through the whole reaction. On the other hand, considering direct FA oxidation, it has been proposed that the unique synergy between platinum (Pt) and palladium (Pd), specifically, the interaction between these metals can result in a more favorable electronic environment for the activation of C–H bonds compared to monometallic catalysts.^54^ The increased activity is believed to arise from several factors, namely, electron redistribution, support effect, improved adsorption properties, site geometry, etc., which remain under ongoing research, as the precise roles of each metal and the nature of their interactions in catalysis remain complex and not fully understood.

This synergy between Pt and Pd may thus offer advantages over monometallic catalysts in catalytic applications, particularly those sensitive to CO poisoning, where the enhanced performance and stability of the bimetallic system could provide better long-term catalytic activity. In the case of Pt–Pd bimetallic NPs, it is likely that a synergistic effect could arise from the complementary nature of the Pt and Pd sites. For instance, the strong interaction of CO with Pt_iso_ species and the relatively weaker interaction with Pd^0^ species could result in cooperative catalytic behavior, where the two metals work together to enhance CO adsorption and activation. Pd could potentially stabilize certain reaction intermediates or assist in the electronic tuning of Pt, promoting more efficient CO/FA intermediates oxidation. This synergy may be further manifested in the interfacial interactions between Pt and Pd atoms, where the electronic structure of Pt can be influenced by the presence of Pd, thereby altering its catalytic activity. Overall, the ability to tune the electronic properties of Pt and Pd through bimetallic interactions could lead to improved catalytic performance for reactions such as CO oxidation, where the cooperation between these two metals plays a pivotal role in enhancing reaction rates and selectivity.

### 3.2 Electrochemical Performance of the Pd_0.64_Pt_0.36_ Catalyst

Figure 6a shows cyclic voltammograms (CV) of the Pd_0.64_Pt_0.36_ catalyst for successively increasing the reversal potential in the anodic scanning direction. Regardless of the value of the anodic reversal potential, a region of hydrogen adsorption/desorption is intact and visible in the potential range of 0.0 to −0.20 V vs Ag/AgCl. Surface oxide formation is visible for the anodic scanning direction and begins at ∼0.70 V vs Ag/AgCl and continues up to the potential corresponding to the oxygen evolution.^37^ A cathodic peak associated with the surface oxide electroreduction is observed during the cathodic potential sweep. The electrochemically active surface area (ECSA) evaluates the catalyst activity. ECSA cannot be determined from the adsorption/desorption charge of hydrogen atoms measured by cyclic voltammetry because, for Pd, surface and bulk hydrogen adsorption occurs simultaneously, and distinguishing between the two processes is not possible from single CV measurements.^37^ Therefore, the electroreduction of Pt and Pd surface oxides forming a monolayer on a catalyst surface is commonly utilized for ECSA determination. However, the surface oxide formation depends on the electrode potential.^55^ It was postulated, based on the angle-resolved X-ray photoelectron spectroscopy study, that a single monolayer of surface oxide is formed on the platinum in 0.5 M H_2_SO_4_ at a potential range of 0.8–1.1 V vs Ag/AgCl.^56^

**Figure 6:**
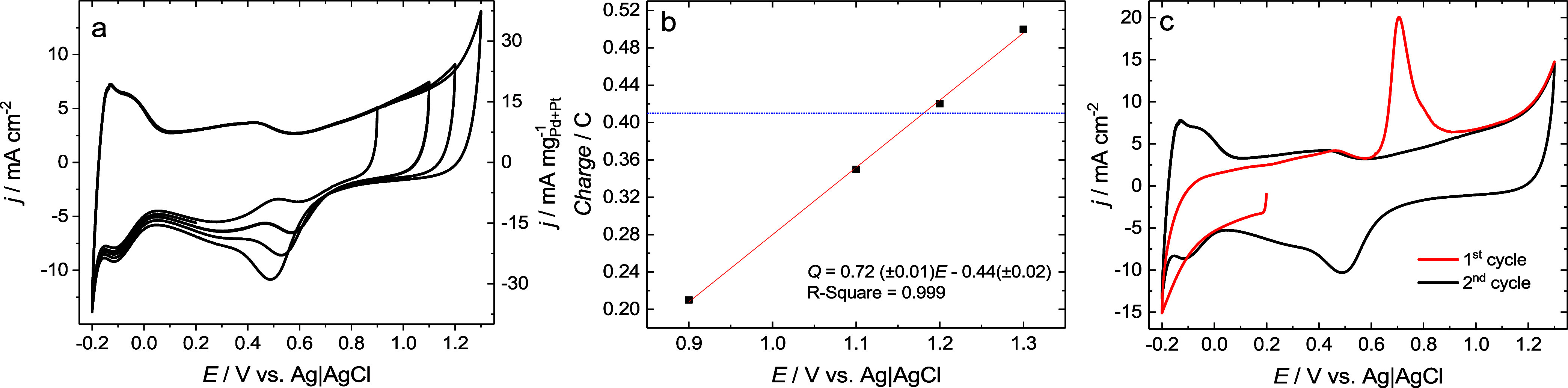
(a) Cyclic voltammetry curves of the Pd_0.64_Pt_0.36_ catalyst in 0.5 H_2_SO_4_ at a scan rate of 10 mV/s. (b) Dependence of the charge associated with the electroreduction of Pt and Pd surface oxides on the anodic turning potential. The blue dashed line shows the value of the charge related to the electrooxidation of CO adsorbed on the catalyst surface. (c) CO-stripping voltammetry of the Pd_0.64_Pt_0.36_ catalyst in 0.5 H_2_SO_4_ at a scan rate of 10 mV/s.

Our results show that with the increase of the reversal anodic potential, the cathodic peak for the oxide electroreduction increases and shifts toward less negative potentials (Figure 6a). It indicates that a more extended or thicker surface oxide film has been formed. The surface oxide charge density was determined by integrating the cathode current for surface oxide electroreduction and plotted as a function of the reversal anodic potential (Figure 6b). The linear increase in charge with increasing reversal potential suggests the continuous formation of surface oxides without a clear boundary between individual monolayer formations. Therefore, CO-stripping voltammetry was additionally used to precisely determine both ECSA and the reversal potential at which a single monolayer of Pt and Pd surface oxides forms (Figure 6c). A sharp anodic peak at ∼0.7 V vs Ag/AgCl is associated with the stripping of adsorbed CO from the catalyst surface. The CO charge (*q*_CO_) was determined by integrating the anode current for CO electrooxidation and used for the ECSA_CO_ determination. The value of *q*_CO_ was indicated as a blue dotted line in Figure 6b. The ECSA_CO_ of the Pd_0.64_Pt_0.36_ catalyst was 56.94 m^2^/g for the formed surface Pt and Pd oxide monolayer at a reversal anodic potential of ∼1.18 V vs Ag/AgCl. Taking into account that the geometric surface area (*S*) of Pd_0.64_Pt_0.36_ nanoparticles is equal to 57,15 m^2^/g (see details in the Supporting Information), one can determine the utilization degree (DU) of the geometric surface of metal nanoparticles using eq 1

1 $$\mathrm{DU}=\frac{\mathrm{ECSA}\times 100}{S}$$

Our results show that almost the entire geometric surface area of the Pd_0.64_Pt_0.36_ catalyst is electrochemically accessible because DU = 99%. The geometric surface area of the Pd_0.64_Pt_0.36_ nanoparticles was determined using eq S1, assuming that the metal particles are spherical in shape. However, HRTEM studies have shown that they are more likely to be icosahedron or cuboctahedron in shape, with a surface area about 20% larger than that of spherical nanoparticles. This means that the DU for such nanoparticles should be about 80%.^57^ On the other hand, the calculation of the available metal surface in the crystallite (S) does not take into account the inaccessibility of part of the crystallite surface due to its contact with the substrate. It follows that the DU for such supported nanoparticles is higher than 80%.

Both the high ECSA value and the availability of active sites reflect the high activity of the Pd_0.64_Pt_0.36_ catalyst toward formic acid electrooxidation. Figure 7a shows the electrocatalytic performance of two catalysts of different Pd-to-Pt ratios (Pd_0.64_Pt_0.36_ versus Pd_0.5_Pt_0.5_), and a commercial catalyst 20 wt % Pd_0.5_Pt_0.5_/Vulcan XC-72 Premetec (0.50:0.50 at. ratio in the metal phase) with 0.6 mg_metal_/cm^2^ loading performed for the typical three-electrode system. The commercial reference catalyst will be briefly referred to as Pd_0.50_Pt_0.50_ ref.

**Figure 7:**
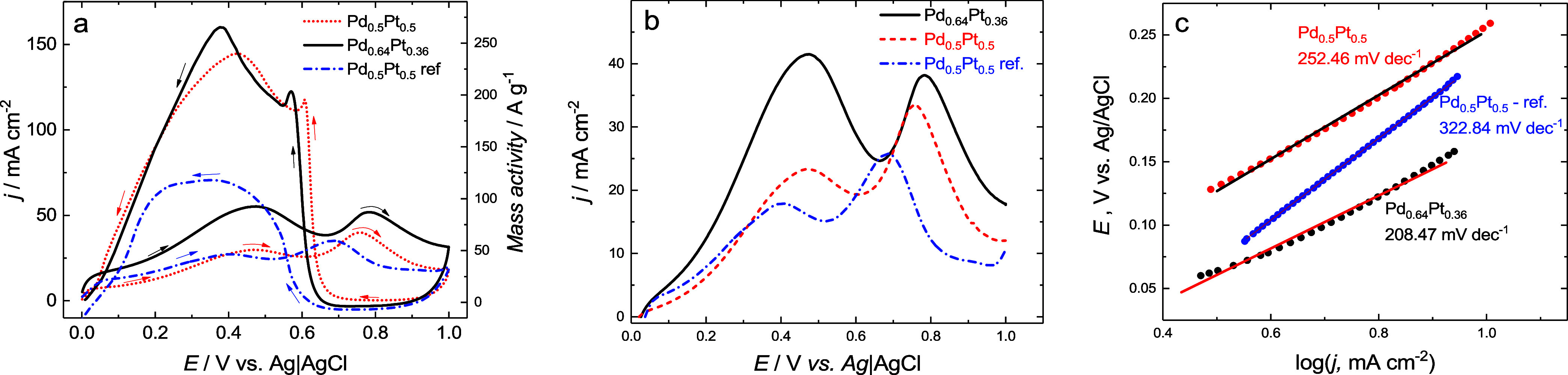
(a) Cyclic voltammetry curves, (b) linear sweep voltammetry curves, and (c) corresponding Tafel plots of Pd_0.64_Pt_0.36_ (black), Pd_0.50_Pt_0.50_ (red), and Pd_0.50_Pt_0.50_ ref (blue) for the 0.5 M HCOOH and 0.5 M H_2_SO_4_ mixed solvent solution recorded at a potential scan rate of 50 mV/s.

The change in the Pd-to-Pt ratio influences the anodic peak currents, signifying the change in the reaction pathway. For all catalysts, there are two peaks on the anodic CV branch, which occur at 0.47 and 0.78 V for the Pd_0.64_Pt_0.36_ catalyst, 0.47 and 0.76 V for Pd_0.50_Pt_0.50_, and 0.39 and 0.69 V for Pd_0.50_Pt_0.50_ ref. The first anodic peak (*j*_HCOOH_) corresponds to the direct oxidation of HCOOH to CO_2_, while the second peak (*j*_CO_) is attributed to the stripping of CO_ads_. CO is in situ generated and adsorbed on the metallic surface according to the dehydration pathway.^58^ Interestingly, for the Pd_0.64_Pt_0.36_ catalyst, the first anodic peak is much higher and broader than that for both Pd_0.50_Pt_0.50_ and Pd_0.50_Pt_0.50_ ref. Moreover, the ratio of the first-to-second anodic peak current for Pd_0.64_Pt_0.36_ is higher than that for Pd_0.50_Pt_0.50_ and Pd_0.50_Pt_0.50_ ref (1.33 to 0.73, and 0.77), indicating that Pd_0.64_Pt_0.36_ exhibits both higher activity toward HCOOH electrooxidation and higher resistance to CO_ad_ surface poisoning. This indicates the dominance of the direct dehydration pathway of FAOR on the Pd_0.64_Pt_0.36_ catalyst, while for Pd_0.50_Pt_0.50_ and the commercially available catalyst, the dehydrogenation pathway dominates. The dominance of the dehydration pathway, while dehydrogenation is also present, may indicate the high activity of the catalyst and its ability to purify the catalyst surface from CO_ad_ and thus prevent the blocking of active Pd/Pt sites. The onset potential for the HCOOH electrooxidation is similar for all catalysts and equal to ∼0.11 V vs Ag/AgCl. Comparable onset potential values were obtained for Pt_0.47_Pd_0.53_.^58^ The lowest Tafel slope shown in Figure 7c indicates that the Pd_0.64_Pt_0.36_ catalyst has a higher activity for FAOR than the other two catalysts. The in situ generated CO_ad_, when adsorbed on the catalyst surface, effectively inhibits the electrooxidation of formic acid. Removal of CO from the catalyst surface by way of electrooxidation of CO_ad_ releases surface sites for subsequent direct oxidation of HCOOH. This is seen during cathodic potential scanning toward lower potentials. This can be observed in the form of a sharp anodic peak characteristic of surface processes of final CO_ad_ removal at 0.57 and 0.61 V vs Ag/AgCl for Pd_0.64_Pt_0.36_ and Pd_0.50_Pt_0.50_, respectively. Similar behavior was observed for the 3-(*N,N*-dimethyldodecylammonio) propanesulfonate-stabilized Pt_0.50_Pd_0.50_/C catalyst^59^ and Pd–Pt alloy.^60^ The cathodic shift of this sharp peak suggests easier removal of adsorbed CO due to weaker binding to the catalyst surface. Immediately following this peak, a large anodic peak for HCOOH electrooxidation is visible at 0.36 and 0.42 V vs Ag/AgCl for Pd_0.64_Pt_0.36_ and Pd_0.50_Pt_0.50_, respectively. Such behavior is not apparent for a commercial catalyst, for which a single broad peak of much smaller amplitude is visible. Notably, both the negative shift in the potential of the HCOOH electrooxidation peak and the increase in the current of this peak demonstrate high activity toward the HCOOH electrooxidation of the Pd_0.64_Pt_0.36_ catalyst. This performance is further confirmed in the DFAFC study.

Figure 8 shows the performance of the Pd_0.64_Pt_0.36_ and Pd_0.50_Pt_0.50_ catalysts as an anode in a DFAFC cell. A long-term stability study measured for 3 M HCOOH, at 50 mA/cm^2^ and 60 °C, with the use of an airflow of 200 mL/min, showed excellent durability of both catalysts (Figure 8a). Interestingly, even after 10 h of cell operation, an increase in voltage was still observed, indicating that the catalysts activate during stability measurements and reach very high activity after 14 h.

**Figure 8:**
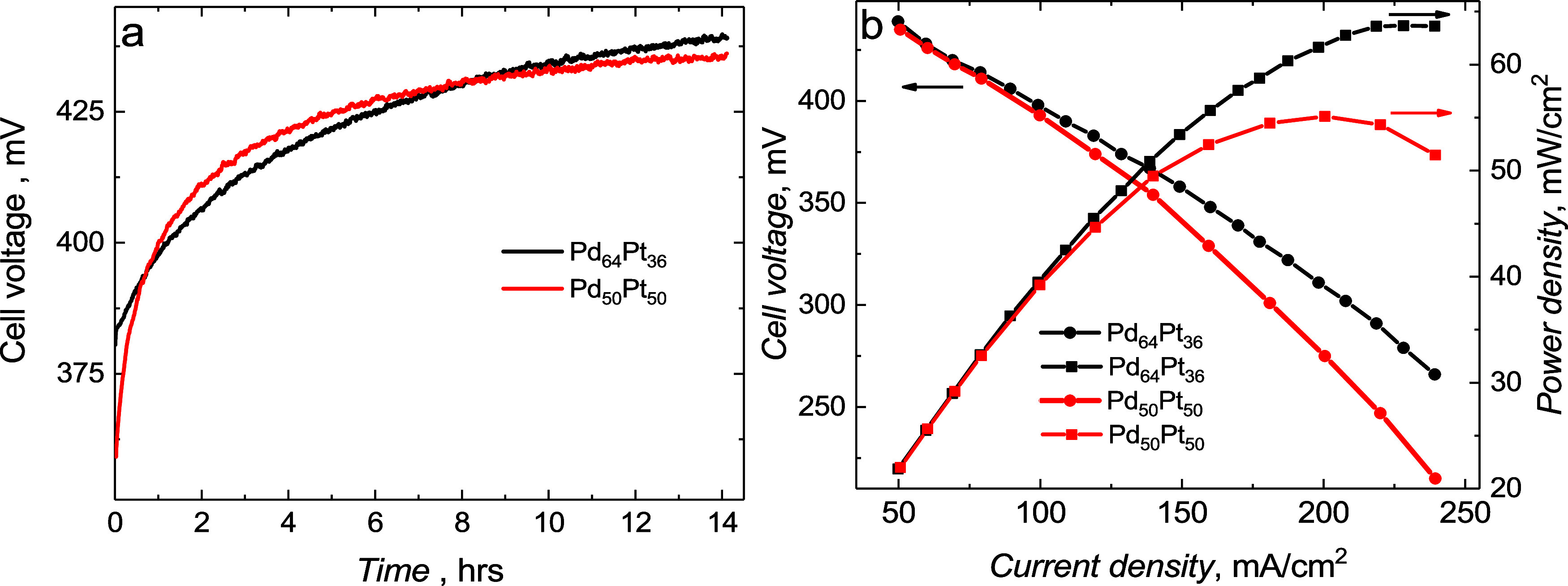
(a) Long-term stability study at 500 mA/cm^2^ and (b) polarization curves and output power of single cell DFAFC of anodic Pd_0.64_Pt_0.36_ and Pd_0.50_Pt_0.50_ catalysts (0.6 mg_metal_/cm^2^) for 3 M HCOOH, at an operating temperature of 60 °C, 4.0 mg_Pt_/cm^2^ as the cathode, and airflow 200 mL/min. Activity and power density measurements were performed after a 14 h stability study (a).

Figure 8b shows the measurement of the cell voltage as a function of current density. For a lower current density, the two catalysts perform similarly, but for a higher current load on the cell, the DFAFC with Pd_0.64_Pt_0.36_ catalysts outperforms the DFAFC with the Pd_0.50_Pt_0.50_ catalyst. This can be attributed to the higher catalytic activity and stability of Pd_0.64_Pt_0.36_, which was also shown in CV measurements. Finally, DFAFC with the Pd_0.64_Pt_0.36_ catalyst shows an excellent power density value of 64 mW/cm^2^ at 250 mA/cm^2^ recorded for airflow on the cathode and 60 °C temperature, while for the Pd_0.50_Pt_0.50_ catalyst, only 53 mW/cm^2^ at 200 mA/cm^2^ was obtained. The results obtained show that the power density obtained for DFAFCs with an anodic Pd_0.64_Pt_0.36_ catalyst is higher (or comparable) to literature results but at metal densities several times lower than the reported metal loadings in the literature (Table 1).

**Table 1:** Comparison of Power Densities Obtained on Pd_*x*_Pt_1–*x*_ Anode Catalysts in a DFAFC Cell

			metal loading (mg/cm^2^)				
catalyst (anode)	power density (mW/cm^2^)	temperature (°C)	anode	cathode	oxidant	fuel	refs
Pd_0.5_Pt_0.5_	64.7@200 mA/cm^2^	room temp.	4	5 (Pt black)	air	9 M	^61^
Pd_0.75_Pt_0.25_	49@243 mA/cm^2^	70	2	2 40 wt % Pt/C	dry O_2_	3 M	^36^
Pd_0.5_Pt_0.5_	5@20 mA	room temp.	1	1 40 wt % Pt/C	air	5 M	^27^
Pd_0.65_Pt_0.35_	64@250 mA/cm^2^	60	0.6	4 (60% Pt/C)	air	3 M	this work
Pd_0.50_Pt_0.50_	53@200 mA/cm2	60	0.6	4 (60% Pt/C)	air	3 M	this work

## 4 Conclusions

Anodic Pd–Pt catalysts for DFAFC were synthesized by using an original, facile, and “green” electrochemical method. A thin layer of a mixture of metal precursors (PtCl_4_ and Pd­(OAc)_2_) with Nafion and MWCNTs was deposited on the surfaces of the carbon cloth in a manner that ensured the effective feeding of the anode with 3 M FA. Pd and Pt nanoparticles were then directly formed on the surface of MWCNTs by electrochemical reduction of precursors. The obtained Pd_0.64_Pt_0.36_ catalysts showed exceptional catalytic activity and high stability toward FAOR on the DFAFC anode. A high power density of DFAFC (64 mW/cm^2^ at 250 mA/cm^2^) was achieved after 14 h of catalyst operation with anodic Pd_0.64_Pt_0.36_ catalyst of 0.6 mg_metal_/cm^2^ loading for 3 M HCOOH, at an operating temperature of 60 °C and a cathode airflow rate of 200 mL/min. A slightly lower power density was obtained by using an anodic Pd_0.5_Pt_0.5_ catalyst under the same measurement conditions. The obtained power density is higher (or comparable) to the literature results for Pd_x_Pt_y_ catalysts. However, it should be noted that for the Pd_0.64_Pt_0.36_ catalyst, several times lower metal densities were used at the anode of the DFAFC cell than those reported in the literature.

The excellent catalytic activity of the Pd_0.64_Pt_0.36_ catalyst can be attributed to the high utilization of its active sites, improved specific activity, and high stability of the Pd_*x*_Pt_1–*x*_ metal particles. The high utilization of active sites of Pd_0.64_Pt_0.36_ catalysts can be explained by the fact that the reported catalysts are deposited as thin films on the surface of the carbon cloth, so that the degree of metal utilization is not limited by the diffusion effects of the reactants. Moreover, the method used for electrochemical fabrication of NPs allows obtaining small (<5 nm) metal crystallites (TEM and XRD results) in a “clean” way, i.e., it does not require the use of chemical additives that can adsorb on the metal surface and consequently reduce the metal utilization rate. HRTEM studies have shown that metal nanoparticles do not form agglomerates on the surface of MWCNTs, which are also effectively dispersed, undoubtedly increasing the accessibility for reactants.

Monometallic Pd catalysts are characterized by having a very high initial activity but are quickly deactivated due to the deposition of CO on the metal surface, which is a byproduct of the FA electrooxidation reaction. In contrast, Pt catalysts possess a much lower initial activity but show high stability due to their ability to electrooxidize adsorbed CO. Therefore, the effective contact between the Pd and Pt phases allows the Pd surface to be cleaned of CO by permeation of CO from Pd to Pt, on the surface of which CO is removed by electrooxidation. Thus, these catalysts achieve enhanced activity and stability due to the synergy of Pd’s high activity and Pt’s stability in the FAOR. The developed Pd–Pt catalyst directly electrodeposited on MWCNTS/carbon paper possesses great potential for use as the anode of direct formic acid fuel cells.

## Supplementary Material



## Notes

Photo of an anode composed of carbon cloths with deposited ink containing surface-modified MWCNTs (∼53.8 wt %), PtCl_4_ (∼13.8 wt %), Pd­(OAc)_2_ (∼14.6 wt %), and Nafion (∼17.8 wt %), a single fuel cell photo, SEM and EDS results, table of model parameters calculated from the XRD measured patterns, XPS survey spectrum, table of the metallic composition of the Pd_0.64_Pt_0.3_ catalyst determined from XPS results, geometric surface area calculations, and table comparing the FAOR activity of the as-made Pt_0.37_Pd_0.63_ catalyst with other reported Pt–Pd-based catalysts (PDF)
