# Supplementary material for: Codeposited Bimetallic Pt–Pd Catalyst Supported on MWCNTs/Carbon Cloth as an Efficient DFAFC Anode Material
Source: ACS Sustain Chem Eng. 2025 Jun 18;13(25):9609–20. doi: 10.1021/acssuschemeng.5c02346 (PMC12217096; doi:10.1021/acssuschemeng.5c02346)
Supplement: Supplementary file 1 [file sc5c02346_si_001.pdf]

Co-deposited bimetallic Pt-Pd catalyst supported on MWCNTs/carbon cloth as an efficient DFAFC anode material

Karol Juchniewicz,[a] Izabela S. Pieta,[a] Bogusław Mierzwa,[a] Marcin Pisarek,[a] Ravishankar G. Kadam,[b, c] Olena Mozgova,[a] Marcin Holdynski,[a] Artur Malolepszy,[d] Andrzej Borodzinski,[a,*] Piotr Pieta[a,*]

[a]Institute of Physical Chemistry, Polish Academy of Sciences, Kasprzaka 44/52, 01-224 Warsaw, Poland

[b]Nanocatalysis Research Laboratories, Czech Advanced Technology and Research Institute (CATRIN), Palacky University, Olomouc, Šlechtitelů 11, 783 71 c, Czech Republic

[c]Nanotechnology Centre, Centre for Energy and Environmental Technologies, VŠB–Technical University of Ostrava, 17. listopadu 2172/15, 708 00, Ostrava-Poruba, Czech Republic

[d]Warsaw University of Technology, Faculty of Chemical and Process Engineering, Warynskiego 1, 00-645 Warsaw, Poland

*Corresponding author's E-mail addresses: A. B. borodzinskiandrzej@gmail.com; P.P. ppieta@ichf.edu.pl

TEL: +48 22 343 32 17, FAX: +48 22 343 33 33

Number of Pages: 5

Number of Figures: 4

Number of Tables: 5

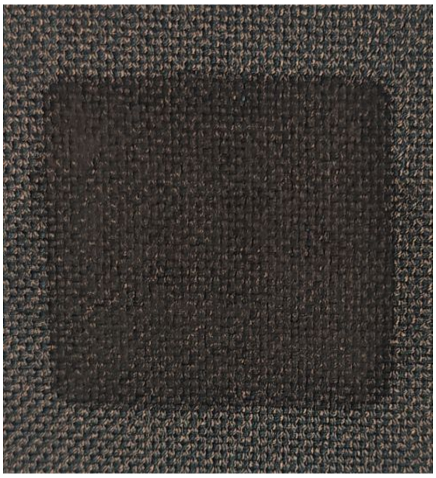

Figure S1. Photo showing carbon cloths with deposited ink containing surface-modified MWCNTs (~53.8% wt.), $PtCl_4$ (~13.8% wt.), $Pd(OAc)_2$ (~14.6% wt.), and Nafion (~17.8% wt.).

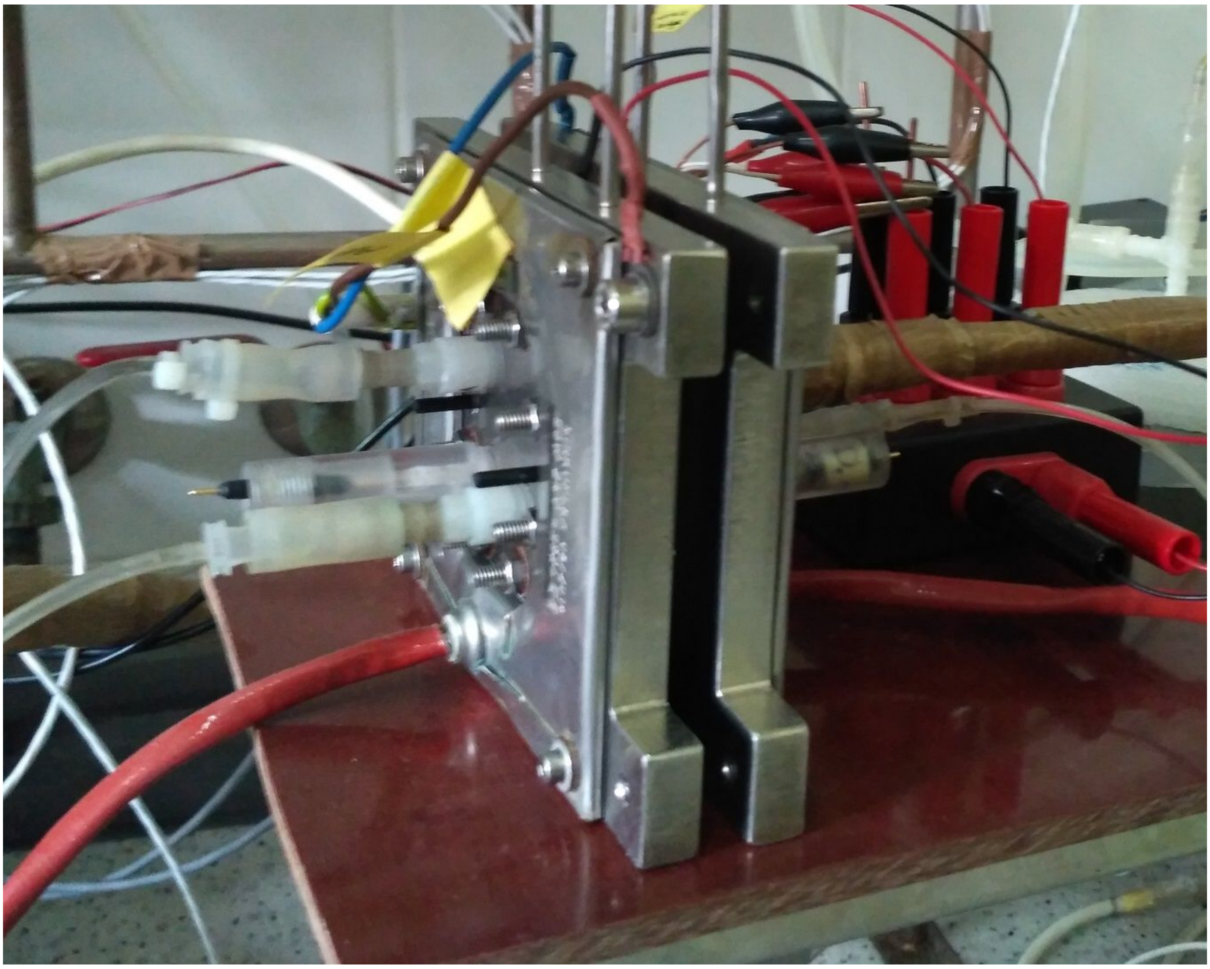

Fig. S2. Photo showing a single fuel cell used to measure the activity and stability of an anode constructed from $Pd_{0.64}Pt_{0.36}$/MWCNTs/carbon cloth catalyst.

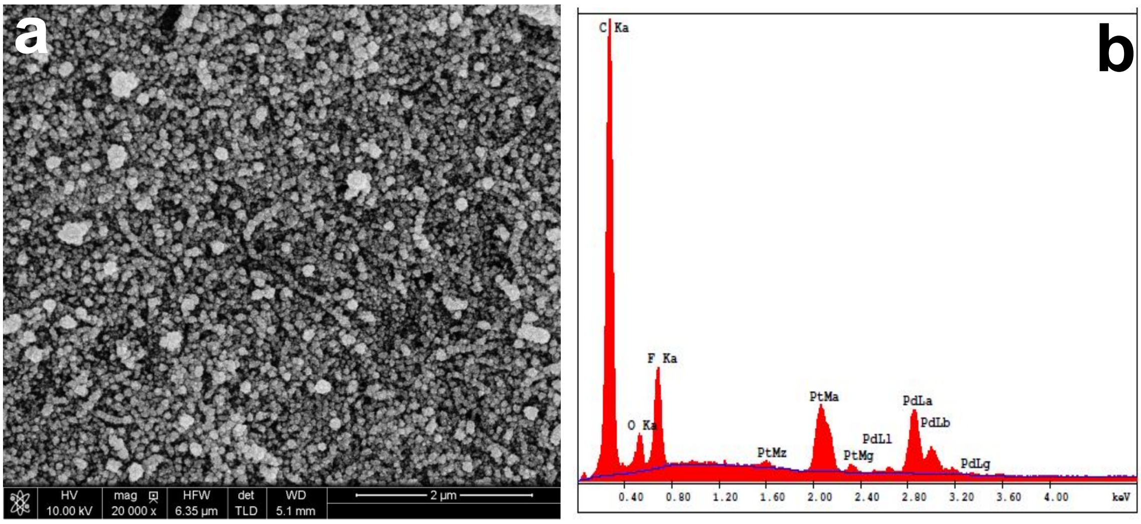

Figure S3. (a) SEM image of the $Pd_{0.65}Pt_{0.35}$ catalyst, (b) corresponding EDX data showing elemental analyses of the sample shown in (a).

Table S1. The elemental composition of the $Pd_{0.64}Pt_{0.3}$ catalyst shown in Fig. S3 calculated from EDX measurements.

Element	Wt %	At %	K-Ratio	Z	A	F
C K	49.77	80.42	0.3406	1.1212	0.6103	1.0001
O K	4.67	5.66	0.0150	1.0953	0.2938	1.0002
F K	8.24	8.42	0.0337	1.0263	0.3980	1.0000
PtM	15.78	1.57	0.1180	0.6862	1.0904	1.0001
PdL	21.55	3.93	0.1675	0.7780	0.9991	1.0000
Total	100.00	100.00				

Element	Net Inte.	Bkgd Inte.	Inte. Error	P/B
C K	978.84	15.19	0.52	64.45
O K	81.80	24.06	2.25	3.40
F K	222.09	33.71	1.24	6.59
PtM	217.49	36.45	1.27	5.97
PdL	219.29	33.53	1.25	6.54

Table S2. Model parameters calculated from the measured patterns, where 2*Θ* is the center position of the peak, *d* is the interplanar spacing, *a* is the lattice parameter/constant and *D* is the estimated average size of the nanoclusters in [hkl] direction.

	Indices	Hydrogen				Air			
	hkl	2Θ / °	d / Å	a / Å	D / nm	2Θ / °	d / Å	a / Å	D / nm
Pd-rich phase	111	38.81	2.32	4.02	7	40.11	2.25	3.89	4
	200	45.12	2.00	4.02	6	46.55	1.95	3.90	5
	220	65.72	1.42	4.02	6	68.12	1.38	3.89	4
Pt-rich phase	111	39.83	2.26	3.92	5	39.8	2.27	3.92	8
	200	46.32	1.96	3.92	4	46.29	1,96	3.92	7
	220	67.59	1.39	3.92	5	67.67	1.38	3.92	7

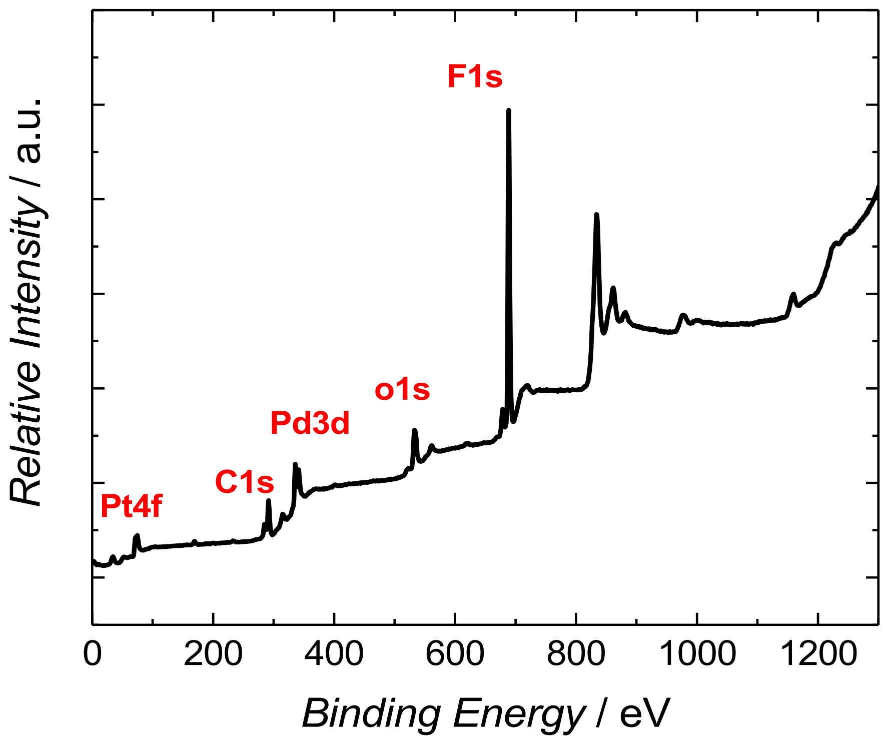

Figure S4. X-ray photoelectron spectroscopy survey spectrum for the $Pd_{0.64}Pt_{0.36}$/MWCNTs catalyst.

Table S3. The metallic composition of the $Pd_{0.64}Pt_{0.3}$ catalyst determined by XPS

Name	Peak BE	Atomic %
Pt4f7	70.4	27.9
Pt4f7	71.9	9.9
Pd3d5	334.8	31.6
Pd3d5	335.8	19.2
Pd3d5	337.3	7.0
Pd3d5	339.2	4.5
		**100.0**

Table S4. FTIR bands assignment for CO adsorbed over Pt, Pd and $Pd_{0.64}Pt_{0.36}$.

Wavenumber, cm^{-1}	assignment/functional group
2215–2195	Pd^{3+}–CO
2215–2140	Pd^{2+}–CO
~2100	Pd^{0}–CO
□2115	CO on $Pd_{iso,}$ CO on $Pt_{iso,}$ CO on the MWCNTs
>2100	supported cationic Pt species (Pt_{ox} clusters, Pt_{iso} species, Pt coordinated with oxidizing ligands)
2077	well-coordinated (WC) Pt sites
□2060	on-top Pt (111)
2058	under-coordinated (UC) Pt sites
1716<br>1698	C=O (keto C double bond O modes), bridge bonded CO

The geometric surface area of $Pd_{0.64}Pt_{0.36}$ nanoparticles was determined using Eq. S1.

$$S = \frac{6000}{\gamma_{PdPt} \cdot d_{PdPt}} \qquad \text{(S1)}$$

where S is the surface area (m^2/g), d_{PdPt} = 5 nm the average particle size from (220) XRD peak, γ is the average density of the metallic phase

$$\gamma_{PdPt} = wt\%_{Pd} \cdot \gamma_{Pd} + wt\%_{Pt} \cdot \gamma_{Pt} \qquad \text{(S2)}$$

where *wt*% means weight percent of Pd and Pt equal to $wt\%_{Pd}$=45,9% and $wt\%_{Pt}$=54,1%, γ_{Pd} = 12,9 g/cm^3, γ_{Pt} = 21,4 g/cm^3.

Table S5. Comparison of the FAOR activity of the as-made $Pt_{0.37}Pd_{0.63}$ catalyst with other reported Pt-Pd-based catalysts.

Catalyst	Metal loading mg/cm^2	$ECSA_{CO}$ m^2/g^1	Electrolyte	Catalytic activity mA/cm^2	I_f/I_b	Onset Pot. (V)	References
$Pt_{0.3}Pd_{0.7}$	-	53.6	0.5 M H_2SO_4 0.5 M FA	25.3	1.12	0.03 vs SCE	1
$Pt_{0.5}Pd_{0.5}$	-	62.5	0.5 M H_2SO_4 0.5 M FA	10.7	3.06	-0.1 vs SCE	1
$Pt_{0.62}Pd_{0.38}$/Ti	-	-	0.5 M H_2SO_4 0.5 M FA	12	0.28	0.1 vs SCE	2
$Pt_{0.50}Pd_{0.50}$/Ti	-	-	0.5 M H_2SO_4 0.5 M FA	40	1.33	0.1 vs SCE	2
Pt@Pd/C	0.042	156.5	0.5 M H_2SO_4 2 M FA	60	3	-0.1 vs Ag/AgCl	3
$Pt_{0.47}Pd_{0.53}$	0.43	32	0.1 M $HClO_4$ 0.1 M FA	4.14	0.15	0 vs SHE	4
$Pd_{0.75}Pt_{0.25}$	0.2	49.6	0.5 M H_2SO_4 1 M FA	59.34	0.4	0.278 vs Ag/AgCl	5
$Pt_{0.37}Pd_{0.63}$	0.6	56.94	0.5 M H_2SO_4 0.5 M FA	52	1.17	0.15 vs Ag/AgCl	This work

(1) Zhao, Q.; Ge, C. W.; Cai, Y.; Qiao, Q. C.; Jia, X. P. Silsesquioxane stabilized platinum-palladium alloy nanoparticles with morphology evolution and enhanced electrocatalytic oxidation of formic acid. *J Colloid Interf Sci* **2018**, *514*, 425-432. DOI: 10.1016/j.jcis.2017.12.053.

(2) Yi, Q. F.; Huang, W.; Liu, X. P.; Xu, G. R.; Zhou, Z. H.; Chen, A. C. Electroactivity of titanium-supported nanoporous Pd-Pt catalysts towards formic acid oxidation. *J Electroanal Chem* **2008**, *619*, 197-205. DOI: 10.1016/j.jelechem.2008.03.012.

(3) Wu, Y. N.; Liao, S. J.; Su, Y. L.; Zeng, J. F.; Dang, D. Enhancement of anodic oxidation of formic acid on palladium decorated Pt/C catalyst. *J Power Sources* **2010**, *195* (19), 6459-6462. DOI: 10.1016/j.jpowsour.2010.04.062.

(4) Ghosh, S.; Raj, C. R. Pt-Pd nanoelectrocatalyst of ultralow Pt content for the oxidation of formic acid: Towards tuning the reaction pathway. *J Chem Sci* **2015**, *127* (5), 949-957. DOI: 10.1007/s12039-015-0854-6.

(5) Muthukumar, V.; Chetty, R. Electrodeposited Pt-Pd dendrite on carbon support as anode for direct formic acid fuel cells. *Ionics* **2018**, *24* (12), 3937-3947. DOI: 10.1007/s11581-018-2526-2.
